# P022 Catastrophic antiphospholipid syndrome: early recognition of a rare and life threatening presentation

**DOI:** 10.1093/rap/rkag113.052

**Published:** 2026-11-03

**Authors:** Samuel McCormick, Mark Leith

**Affiliations:** South Eastern Health and Social care trust, Belfast, United Kingdom; South Eastern Health and Social care trust, Belfast, United Kingdom

## Abstract

**Introduction:**

Antiphospholipid syndrome is a systemic autoimmune disorder characterised by thrombotic events and recurrent miscarriage. Although thrombosis is the hallmark of the disease, non-thrombotic manifestations affecting the kidneys, heart and other organs are increasingly recognised. Here is a case of catastrophic APS who has had recurrent admissions with hyper-inflammatory states and a range of complications including myocarditis, endocarditis, renal impairment and HLH. This case highlights the potential for profound immune dysregulation in APS and the role of targeted immunoregulatory therapy in achieving disease control.

**Case description:**

This 44-year-old male with a history of previous DVT and PE presented to hospital in February 2022 with non-specific viral symptoms. He was anaemic with a haemoglobin of 75 g/L. Subsequent pyrexias, splenomegaly, thrombocytopenia and high ferritin levels led to a bone marrow biopsy confirming haemophagocytic lymphohistiocytosis (HLH). He had proteinuric renal impairment. He was strongly triple positive for antiphospholipid antibodies. On the same admission he had rising troponin T and BNP levels. CT-PET scan and echocardiogram showed evidence of myocarditis resulting in initiation of steroid therapy in conjunction with cardiology. Steroids were weaned and he remained well for several years.

In July 2025 he was admitted with vomiting, diarrhoea and night sweats. He was found to have a large vegetation on his aortic valve and treated as presumed infective endocarditis, however cultures were negative, and there was a lack of response to IV antibiotics. He was subsequently diagnosed with Libman-Sacks endocarditis secondary to APS. He developed a further hyper-inflammatory state with HLH features. APS antibodies were negative on this occasion, but this was felt to be consumptive. He again had proteinuric renal impairment and a profound immune-mediated thrombocytopenia with platelets of 3x10⁹/L. ANA and CTD screens were negative. He was treated with high-dose steroids, IV immunoglobulins and rituximab with significant clinical improvement. He was discharged on warfarin, hydroxychloroquine and prednisolone.

On last review in May 2026 he was generally stable overall. His LV function has returned to normal and his vegetation has resolved. He is maintained on hydroxychloroquine and warfarin.

He was followed up by the renal team with concerns for a possible lupus nephritis. Renal biopsy did not show any signs of immune complex disease, however, the biopsy did show focal segmental glomerulosclerosis which can be caused by APS.

**Discussion:**

This patient illustrates a particularly aggressive case of APS, characterised by recurrent episodes of immune hyperactivation rather than isolated thrombotic events. I think this case is interesting because of the wide-ranging manifestations that were found.

Catastrophic APS (CAPS) is a rare but life-threatening form of APS seen in just 1% of APS cases. It is characterised by rapidly progressive, widespread thrombo-inflammation resulting in diffuse small vessel thrombosis and multiorgan failure.

Triggers for Catastrophic APS include viral illness, surgery, trauma, obstetric complications and withdrawal of anticoagulation. In many cases no obvious trigger is identified.

Over 50% of catastrophic APS cases will have cardiac and/or renal involvement. Possible cardiac manifestations include valve disease (typically non infective endocarditis), heart failure, myocardial infarction and myocarditis. APS with renal involvement may cause reduced renal function with associated hypertension, proteinuria and haematuria.

During the patients index admission he had both myocarditis and renal impairment. This was treated with high-dose corticosteroids in conjunction with cardiology. Treatment was escalated during the second admission to include immunoglobulin given the severity of presentation with HLH, renal impairment and non-infective endocarditis. This patients’ immune mediated thrombocytopenia was limiting the team’s ability to anticoagulate so rituximab was started with good effect.

The patient is maintained on hydroxychloroquine as a steroid sparing agent and warfarin to prevent thrombosis.

I have learned about the broad spectrum of presentations that can be caused by APS. This case illustrates this well and emphasises the importance of considering immune mediated complications when patients are unwell with multiple organ involvement. Early recognition and prompt multidisciplinary management may improve outcomes in this rare but potentially life-threatening presentation.

The main question in this case is why this patient is having such severe episodes and what can be done to limit this response to insults such as infection.

**Key learning points:**

• Patients with APS can have severe hyper-inflammatory episodes with multiple organ involvement called catastrophic APS.

• These episodes can be triggered by insults such as infection or surgery.

• Early recognition and prompt testing for antiphospholipid antibodies are essential, as delayed diagnosis can significantly worsen outcomes.

• Awareness of catastrophic APS is critical as it requires urgent, aggressive treatment and carries a higher mortality if untreated.

• Anticoagulation remains the mainstay of treatment, but severe cases may require steroids, immunoglobulins or biologic agents.

**Learning objectives**

• To improve my understanding of the diagnosis and management of complex presentations of APS, particularly in patients with severe or atypical disease.

• To gain expert insight into current evidence and emerging therapies for refractory or high-risk APS, including the role of biologic agents and adjunctive treatments.

• To develop a greater understanding of factors associated with poor prognosis and strategies to prevent recurrent thrombotic events.

• To receive constructive feedback on the clinical reasoning and management decisions in this case to inform my future practice.

• To enhance my knowledge of current research priorities and evidence gaps in APS, particularly regarding optimal management of severe disease.

